# Compulsory notification of injuries in aesthetic procedures. Impact on patient safety^⋆^

**DOI:** 10.1016/j.abd.2022.01.002

**Published:** 2022-05-30

**Authors:** Érico Pampado Di Santis, Samira Yarak, Marcos Roberto Martins, Sergio Henrique Hirata

**Affiliations:** a Evidence-Based Postgraduate Program in Health, Escola Paulista de Medicina, Universidade Federal de São Paulo, São Paulo, SP, Brazil; b Postgraduate Program in Translational Medicine, Department of Medicine, Escola Paulista de Medicina, Universidade Federal de São Paulo, São Paulo, SP, Brazil; c Sector of Pathology, Department of Medicine, Universidade de Taubaté, Taubaté, SP, Brazil

**Keywords:** Aesthetics, Beauty and aesthetics centers, Health impact assessment, Health planning guidelines, Intraoperative complications, Surgery, plastic

## Abstract

The disparity between the number of aesthetic procedures performed worldwide, and the complications described in the literature is remarkable. Doubts regarding the underreporting are reasonable and should be considered. The aim of this study is to demonstrate the scarcity of scientific publications on complications in aesthetic procedures compared to the abundance of these procedures performed worldwide. Based on this knowledge, it will be demonstrated to the health authorities the importance of compulsory notification of complications in aesthetic procedures that require medical attention so that the available data will allow their prevention. The limitation of knowledge regarding complications was demonstrated in the data collection for the preparation of the thesis “Deaths Related to Liposuction in Brazil” presented in 2018 and published in Surgical and Cosmetical Dermatology in 2020. The definition of complication in aesthetic procedures needs to be objective to prevent different and subjective interpretations. With the compulsory notification of complications in aesthetic procedures, it is intended to learn about their causes to develop guidelines for their prevention.

## Aesthetic procedure complication

The coherent and concrete definition of aesthetic procedure complication (APC) is understood as: physical damage caused to the human being resulting from an intervention performed by physicians or non-physicians aiming to modify the physical appearance of the human being and that requires outpatient and/or hospital medical care. In short, it is physical damage caused by a procedure with the objective of modifying physical appearance that develops into an adverse effect and that requires medical care.

According to this definition, APC is not limited to interventions that take place in clinics or hospitals but also in environments licensed by the Health Surveillance where aesthetic procedures are performed.

Death during plastic surgery, exogenous poisoning caused by substances used to modify the hair shaft, contamination or allergy after the introduction of exogenous pigments into the dermis through needles, or blindness after undergoing a facial filler treatment are examples of complications in aesthetic procedures.

## Disparity between the number of procedures performed and the reported complications

The search performed in the Pubmed database in December 2021 using the words: complications AND aesthetic procedures, with a limited time interval between 2018 and 2021, resulted in 3,685 studies. The latest data reported by ISAPS, the International Society of Aesthetic Plastic Surgery,^1^ regarding the procedures performed in 2018 and 2019 were: 48,248,678; of these, 23,266,374 in 2018 and 24,982,304 in 2019, an increase of 7.37%.

The relationship between the performed procedures reported by ISAPS and the number of published complications does not seem to reflect the reality at all, as it suggests that only 0.007% of the procedures have complications or adverse effects. This indicates the existence of underreporting, as it is natural to publish only the most serious, unusual, or severe complications. And here is the origin of one of the problems that the authors want to address.

Moreover, the increase in the number of medical and non-medical professionals who perform aesthetic procedures is noteworthy, and there is reasonable doubt as to how much is known about complications in aesthetic procedures and their probable reality, which can impair effective preventive measures against these complications.

## Identification of complications in aesthetic procedures

Getting to know the complications that occur in aesthetic procedures is not easily accessible information. The data are restricted to confidential medical records, published in scientific studies, or published by the media; in the latter example, most of the time, when the evolution of the complication is fatal or the patient is a public figure. In other cases of complications, the suffered experience is veiled in the patient's individuality. The shame, the family 'members' annoyance in relation to the incident, the money spent on it, and the physical and emotional damages resulting from the treatment prevent the reparation, consequent to the complication, from being claimed.

As a byproduct of data from the thesis “Liposuction-related Deaths in Brazil”^2^, ^3^ presented at *Universidade Federal de São Paulo – Escola Paulista de Medicina* by the Evidence-Based Health Program, the bill (PL) n. 9602/2018 was enacted in the Brazilian House of Representatives. The project proposal is for all complications related to aesthetic procedures to be notified, on a compulsory basis, throughout the country. After a public hearing with representatives of the *Conselho Federal de Medicina* (Federal Council of Medicine), the Brazilian Society of Dermatology and of Plastic Surgery, and the Cochrane Center of Brazil, PL n. 9602/18 was unanimously approved to be sent to the *Comissão de Seguridade Social e Família* (Social Security and Family Commission). In this Commission, the favorable vote of the Representative who was also rapporteur of the project was also unanimously approved. The merits of PL n. 9602/18 have been approved, and now they will undergo constitutional evaluation and, finally, if approved, will become law.

This experience confirms the importance of the common efforts of the scientific community and the government for the consolidation of laws that can safeguard the population. The present paper seeks to demonstrate the attitudes carried out by both the scientific and the political classes aimed at recognizing the complications in aesthetic procedures.

What is sought is the rapid detection of complications in these procedures that an electronic database can be established. Based on this evidence, effective measures to prevent complications related to aesthetic procedures may be taken.

Relevant numbers show that there may be underreporting of complications in aesthetic procedures worldwide. Without knowing the reality associated with the complications of these procedures, actions taken to prevent them become difficult. The approval of the PL and the enaction of the law will make Brazil the first country to take this type of action, which has been already proposed by other researchers when they concluded their published studies.

## Aesthetic procedures: performance × complication

**Figure 1:**
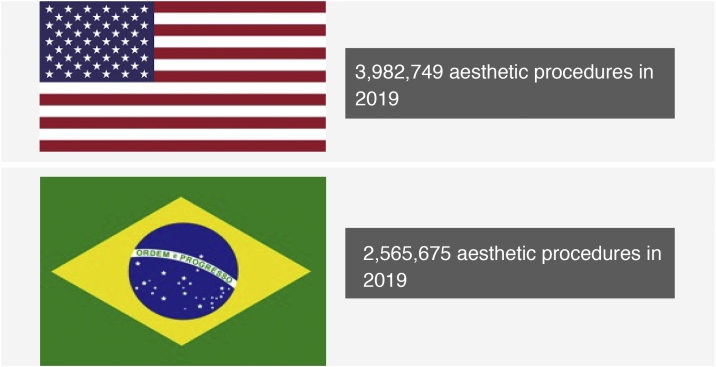
Number of procedures performed in the US and Brazil in 2019. Source: ISAPS.^2^

The numbers of aesthetic procedures are impressive (Fig. 1). According to ISAPS, in 2018, 1,862,506 mammaplasties, 1,732,620 liposuctions and 1,099,960 blepharoplasties were performed, in addition to: 6,097,516 Botox applications and 3,729,833 dermal fillings with hyaluronic acid. In 2019, there was a mean increase of 7.4%, reaching: 1,795,551 mammaplasties, 1,704,786 liposuctions, 1,259,839 blepharoplasties, 6,271,488 Botox applications and 4,315,859 dermal fillings with hyaluronic acid (Table 1).

**Table 1:** Number of aesthetic procedures.^2^

	Mammoplasties	Liposuctions	Blepharoplasties	Botox	Hyaluronic acid fillings
2018	1,862,506	1,732,620	1,099,960	6,097,516	3,729,833
2019	1,795,551	1,704,786	1,259,839	6,271,488	4,315,859
Variation in relation to 2018	-3.6%	-1.6%	+14.5%	+2.9%	+ 15.7%

In Brazil, this same association shows that in 2019, 2,565,675 aesthetic procedures were performed, with the country being second only to the United States, which totaled 3,982,749 aesthetic procedures performed in that year. The numbers may be higher than the statistics show due to the fact that these variables were collected in an organized but restricted association.^2^

All aesthetic procedures are subject to complications. The classification of aesthetic procedures is confusing and lacks a scientific definition. Many terms related to these procedures have been created and lead to the feeling that they are low risk, low complexity, and free from adverse effects. This creates an illusion for the patient and even for the professionals who perform them without an adequate idea of the danger that these procedures can bring. Procedures considered to be “minimally invasive” are not free from complications. They cannot be trivialized nor carried out indiscriminately, putting the health of the population at risk. Despite the minimally invasive aspect, short recovery time, not involving general anesthesia, and hospitalization, they reach deep cutaneous planes. Botox is applied to muscles, and the fillers can be deposited on the periosteal plane.

Even scientific articles use the term “minimally invasive”; however, they do not conceptualize it and only exemplify certain procedures as such: application of Botox, dermal fillers, microdermabrasion, laser, radiofrequency, and ultrasound.^4^, ^5^, ^6^, ^7^

The intention of the PL is not to prohibit or punish professionals, with different training or technical training, from performing aesthetic procedures but to recognize all complications in these procedures so that, based on this irrefutable evidence, plans can be drawn to prevent complications in each one of them. It is a preventive project.

The literature shows us complications after different aesthetic procedures: after tattooing, body piercings, hair straightening, application of Botox, blepharoplasty, facial and body fillings, and liposuction, among others.

In a systematic review published in 2016, Dieckmann et al.^7^ identified 67 cases published between 1984 and 2015 of severe bacterial infection complications after intradermal deposition of exogenous pigments (tattoos). Cases of bacterial endocarditis associated with tattooing and body piercing in young adults aged 15 to 30 years, with and without congenital heart disease, were published by Armstrong et al.^8^ These authors emphasize in their conclusions the importance of the role of an international file of complications related to body art, which would offer documentation that could provide data for the implementation of preventive measures.

Miranda-Vilela et al.^9^ emphasize that the use of chemical products in the hair can cause itching, burns and scars on the scalp and that there should be stricter legislation on these chemical products. Akkaya et al.^10^ studied a case of unilateral mydriasis and eyelid ptosis after an aesthetic procedure with Botox. They report the case of a 36-year-old woman who developed the complication three days after the application.

In aesthetic procedures that use technologies, such as certain types of non-ablative lasers, Handley^11^ states that little has been published regarding complications with these treatments, and it is difficult to obtain data on adverse events; however, he mentions: transient hyperpigmentation and hypopigmentation; permanent hypopigmentation, scarring, infection, 1^st^- and 2^nd^-degree burns, areas of alopecia, folliculitis, pseudofolliculitis, stimulation of hair growth on the periphery of treated areas and temporary acne. Regarding surgical procedures, Lelli and Lisman^12^ carried out a review of the main complications of blepharoplasty. The authors reported complications after the first week of surgery, such as corneal abrasions and retrobulbar hemorrhage, which can lead to blindness, and later on, complications such as strabismus, corneal exposure, and epiphora (loss or alteration of the normal drainage of tears through the lacrimal pathways), changes in the eyelid height and contour, together with asymmetries, scarring, and persistent edema.

**Figure 2:**
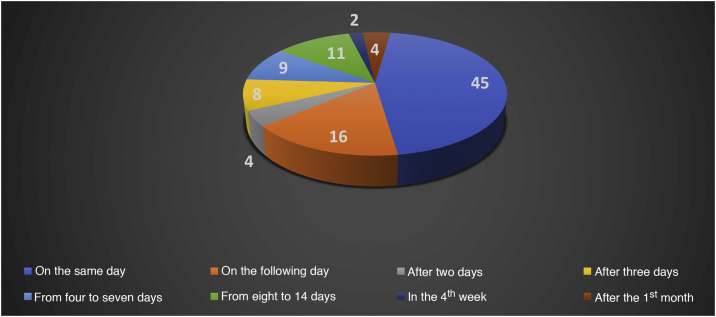
Absolute number of deaths in liposuction in the period from 1987 to 2015.^2^, ^3^

**Figure 3:**
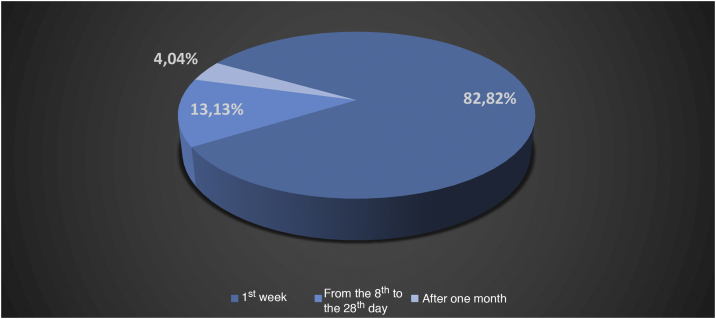
Relative number of deaths in liposuction in the period from 1987 to 2015.^2^, ^3^

Oestreicher and Mehta^13^ reported on abnormalities secondary to lower blepharoplasty and included: lower eyelid retraction (ectropion), lower eyelid contour rounding, rounding of the lateral canthus, and ectropion. Moris et al.^14^ reported complications such as scar dehiscence, skin necrosis, infection, hypoesthesia, and keloids in cervicofacial lifts performed under local anesthesia. Di Santis et al.^3^ reported deaths in liposuction. This study was carried out in Brazil with information collected from written media disclosures. The authors reported that death occurred on the day of the surgery (D0) in 45% of the cases. When considering death between D0 and the end of the first week, D7, this percentage increased to 82.82%, between the 2^nd^ week and the 28^th^ day 13.13%, and 4.04% after the first month (n = 99; Figure 2, Figure 3).^2^, ^3^

A total of 97 institutions were reviewed in which the surgeries were performed. In 53.6% of the cases, they took place in hospitals, and in 46.4%, in clinics outside hospitals. The association of liposuction with other procedures occurred in 38.24% of the cases, while in 61.76%, they were reported as isolated liposuction. Regarding the specialties of the involved physicians, the authors were able to have information in 86 cases. Of these, 66 physicians had a board-certified specialty, 61 in plastic surgery, two in general surgery, two in orthopedics, and one in diagnostic imaging, whereas 20 professionals had no board-certified medical specialty. In 70.93% of the cases, the surgeries were performed by physicians with possible specific training to perform liposuction; 13.64% of physicians were involved in more than one surgery that resulted in the patient death (Table 2).^2^, ^3^

**Table 2:** Causes of death after liposuction, from 1987 to 2015.^15^

		**n**
**Sex**	**Female: 98%**	**102**
**Age**	**Between 31 and 40 years: 40%**	**102**
Death on D0 (day of surgery)	45%	99
Death between D0 and D7	82.82	99
Death after D30	4.04	99
Surgeries in hospitals	53,6%	97
Isolated liposuction	61.76%	107
Specialty with the highest number of deaths	Plastic surgery	86
Number of cases in which the surgeon signed the death certificate	12.98%	86
Physicians involved in more than one death	13.64%	86

Among the causes of death, thromboembolism ranked first, with 17.44% cases, followed by perforation (13.95%), infection (9.3%), hemorrhage (5.81%), fat embolism (4.65%), and acute pulmonary edema and anesthetic complications with 2.32% each (n = 86). In 44.18% of the cases, the cause of death could not be determined.^2^, ^3^

Grazer and de Jong^15^ sent correspondence to the physicians, and the information obtained from the questionnaire regarding the first two causes of death coincided with the report by Di Santis et al.^4^ On the other hand, Lehnhardt et al.^16^ observed that the main causes of death were infection (65%), perforation (13%), followed by thromboembolic phenomena (8%), whereas undetermined cause of death ranked last.^17^ These authors, even with the support of several medical societies and after sending 3,000 questionnaires, accounted for only 23 cases of death.^17^

Cupello et al.^17^ highlighted that their data should be interpreted with caution due to the methodology used, which involved correspondence, with many missing responses. It is therefore observed that the literature itself is contradictory regarding cause of death.^4^, ^16^, ^17^, ^18^

Complications can happen, and, therefore, one needs objective information and knowledge of how many fatalities occurred. It is necessary to quickly diagnose a substance that causes complications before the initiation of an epidemic of cases caused by a product or technology that brings harm. Surgical techniques, sometimes well-established ones, known and desired by patients, can physically and physiologically harm a previously healthy individual and transform him into a sick individual; therefore, they should be reviewed. Action must be taken in cases of physicians and other non-medical professionals who perform highly complex surgical procedures and who consistently make mistakes.

## Actions taken in relation to the topic

Since 1977, the literature has been concerned with complications, with the first article published on abdominoplasty, a very popular procedure in the 1970s.^19^

The Federal Council of Medicine established safety parameters for performing liposuction through Resolution 1711/2003.

The concern with adverse outcomes or complications is an old one. In the House of Representatives, through the Request for Information n. 1.046/1999, the Ministry of Health was asked whether there were available data on the number of deaths related to liposuction in Brazil.

Law n. 12,842/2013 (Medical Act Law), which rules over the practice of Medicine, received a veto regarding items I and II of §4 of the 4^th^ article. This article deals with physicians private activities and §4 deals with invasive procedures. The allegation for the vetoes was that invasive procedures were “(…) broadly and imprecisely” characterized and that there is an extensive list of procedures from a multi-professional perspective.

## Line of reasoning

What we have been able to verify in these years of study and reviewing the literature on complications in aesthetic procedures is that complications can occur as a result of three main factors, with the exception of the inherent human response to the procedure, such as hypersensitivity or auto-inflammatory reactions, and they comprise: the product or technology employed; the utilized technique, many times established and replicated; human error.

In the United States, the states of Florida and Alabama report complications that occur in offices or clinics, not specifically aesthetic procedures, but those that are performed outside the hospital environment when there is death or transfer to a hospital.

In Florida, the data are in public domain, whereas in Alabama, they are not. In the latter state, between 2003 and 2009, 42% of transfers to hospitals were associated with aesthetic procedures. After the introduction of mandatory reporting, there have been no deaths from aesthetic surgeries in Alabama.^19^, ^20^

It is plausible to think that, somehow, there has been an improvement regarding some factors (surgical technique, caution, previous assessment of the patient health, patient monitoring) that culminated in the absolute decrease in death cases in this American state.^20^

In Florida, data that were made available by the Florida Agency for Health Care Administration showed that 63% of the reported deaths were associated with aesthetic procedures.^20^

The notification must be electronic and accessible so that doctors or hospitals can carry it out.

The predicted increase in aesthetic procedures in Brazil draws the attention of health authorities and medical institutions. It is known that aesthetic procedures such as dermal fillings, blepharoplasty, and liposuction, among others, are being performed by non-medical professionals.

To learn about the complications, correlating them to their performers, whether medical or non-medical professionals, with the techniques used, the products, and technologies, is crucial for the scientific knowledge of what can and cannot be performed by non-medical professionals, what can or cannot be performed in relation to surgical techniques, even the well-established ones, and the products or technologies that result in complications. This quick diagnosis, which will be achieved with the compulsory notification, will allow the assesment of the safety of the technique/procedure used for the persons who are submitted to aesthetic procedures.

## Conclusion

Compulsory notification of complications in aesthetic procedures will allow the creation of a database that will facilitate the understanding of their causes and propose possible interventions.

It is important to emphasize that this is not a law aimed at punishment but a means to obtain information with a specific scientific purpose: to identify the causes that led previously healthy individuals who underwent highly elective interventions, often young individuals in the active phase of their lives, to suffer complications. Additionally, to quickly recognize poor quality products or technologies, which can put the health of the population at risk.

## Financial support

FUNADERSP – Fundo de Apoio à Dermatologia do Estado de São Paulo – Sebastião Sampaio.

## Authors' contribution

Érico Pampado Di Santis: Statistical analysis; approval of the final version of the manuscript; design and planning of the study; drafting and editing of the manuscript; collection, analysis, and interpretation of data; effective participation in research orientation; intellectual participation in the propaedeutic and/or therapeutic conduct of the studied cases; critical review of the literature; critical review of the manuscript.

Samira Yarak: Statistical analysis; approval of the final version of the manuscript; design and planning of the study; drafting and editing of the manuscript; collection, analysis, and interpretation of data; effective participation in research orientation; intellectual participation in the propaedeutic and/or therapeutic conduct of the studied cases; critical review of the literature; critical review of the manuscript.

Sérgio Henrique Hirata: Approval of the final version of the manuscript; design and planning of the study; drafting and editing of the manuscript; collection, analysis, and interpretation of data; effective participation in research orientation; intellectual participation in the propaedeutic and/or therapeutic conduct of the studied cases.

Marcos Roberto Martins: Design and planning of the study; drafting and editing of the manuscript; collection, analysis, and interpretation of data; effective participation in research orientation; intellectual participation in the propaedeutic and/or therapeutic conduct of the studied cases.

## Conflicts of interest

None declared.
